# Purified Dietary Aflatoxin B_1_ Does Not Impair Broiler Growth Across a Broad Exposure Range but Elicits Subclinical Biological and Tissue Responses

**DOI:** 10.3390/toxins18100414

**Published:** 2026-09-27

**Authors:** Qiaomin Duan, Wanjun Zhang, Ying Liu, Sunlin Luo, Wenjun He, Wenjun Yang, Yiqiang Chen

**Affiliations:** 1 State Key Laboratory of Animal Nutrition and Feeding, College of Animal Science and Technology, China Agricultural University, Beijing 100193, China; xiaoduo@cau.edu.cn (Q.D.); wanjunzhang@cau.edu.cn (W.Z.); liuying01@cau.edu.cn (Y.L.); luosunlin@cau.edu.cn (S.L.); hewenjun0525@163.com (W.H.); ywj16@sina.com (W.Y.); 2 Ministry of Agriculture and Rural Affairs Key Laboratory of Feed Safety and Biological Efficacy, China Agricultural University, Beijing 100193, China

**Keywords:** aflatoxin B_1_, broiler chicken, oxidative stress, intestinal barrier, bile acid homeostasis, subclinical toxicity

## Abstract

Aflatoxin B_1_ (AFB_1_) toxicity in poultry is often investigated using naturally contaminated ingredients, which can confound its effects with co-occurring mycotoxins and changes in feed quality. Male Arbor Acres broilers received a common basal diet supplemented with purified AFB_1_ at nominal concentrations of 0, 5, 10, 20, 50, 100, or 200 μg/kg for 42 d. The reported analyses detected no significant treatment differences in growth performance or major organ indices. Hepatic antioxidant enzyme activities and total antioxidant capacity decreased at selected exposures, while serum antioxidant enzymes, cytokines, and hematological indices showed selective responses. Jejunal villus height was lower in the nominal 50–200 μg/kg groups. Renal tubular lesions were most evident at 50–100 μg/kg. Bile-acid profiling in four selected groups showed compartment-specific differences. These findings suggest that selected biological and morphological responses may occur without detectable growth impairment under the conditions tested. Isolated biomarker differences remain exploratory, and the data do not establish a formal ranking of endpoint sensitivity or a dietary safety threshold.

## 1. Introduction

Aflatoxin B_1_ (AFB_1_) remains an important feed-safety concern because of its hepatotoxic, immunotoxic, mutagenic, and carcinogenic properties and its continued occurrence in feed ingredients and complete feeds [1]. The liver is a major target organ of AFB_1_ because hepatic bioactivation generates reactive metabolites and promotes oxidative stress, lipid peroxidation, macromolecular damage, and, at sufficiently high exposures, hepatocellular injury [2,3]. AFB_1_-associated oxidative disturbance has also been reported in extrahepatic tissues, including the kidney and intestine, and may be accompanied by alterations in immune and inflammatory signaling [4,5,6,7]. However, much of the mechanistic evidence for overt aflatoxicosis has been obtained at exposure levels that produce clear biochemical, histological, or productive consequences. Less is known about the biological responses that occur during chronic exposure to lower concentrations of AFB_1_ that are more relevant to feed-safety assessment [8].

Chinese feed limits are category-specific: GB 13078-2017 specifies an AFB_1_ maximum of 10 μg/kg for compound feed for young poultry and 20 μg/kg for other compound feeds [9]. The standard does not itself define an age boundary that directly maps these categories onto the two feeding phases used here. Accordingly, 10 μg/kg is considered a young-poultry feed reference, rather than an assumed limit applying uniformly to the entire 42 d experiment. International provisions also differ in their target analyte and animal category. Under EU Directive 2002/32/EC, the maximum AFB_1_ content is 5 μg/kg in compound feed for young poultry and 20 μg/kg for poultry other than young animals, expressed relative to 12% feed moisture [10]. In the United States, the FDA action level is 20 μg/kg for total aflatoxins in relevant feeds for immature animals, including chickens younger than eight weeks, with feed-category exceptions specified in the guidance [11]. These values provide regulatory context rather than interchangeable biological effect thresholds. From a risk-assessment perspective, an important question is whether the absence of impaired growth can be regarded as evidence of biological safety at concentrations close to or moderately above current regulatory limits. Broiler studies have produced inconsistent dose–response relationships. Naturally contaminated or fungal-cultured feed ingredients have been associated with reduced feed intake, impaired weight gain, altered serum chemistry, oxidative stress, and tissue lesions at concentrations ranging from several hundred micrograms per kilogram to the milligram-per-kilogram range, whereas other studies have reported limited effects on growth at comparable nominal exposures [12,13,14,15]. One important source of this variability is the contamination model itself. Naturally mold-contaminated ingredients may contain additional mycotoxins and fungal metabolites and may also undergo deterioration in nutrient quality [16]. Consequently, effects attributed to AFB_1_ cannot always be separated from those caused by co-contaminants or changes in the nutritional characteristics of the feed. Supplementation of purified AFB_1_ into a nutritionally identical basal diet provides a useful approach for defining the dose-dependent biological effects attributable specifically to AFB_1_.

Growth performance is an essential production endpoint, but it may be less sensitive than biochemical, metabolic, or tissue-level indicators of chronic exposure [17]. Oxidative/redox responses, immune mediators, and tissue morphology may change before reductions in body-weight gain or feed efficiency become detectable [18]. The intestine is particularly relevant because it is the first major tissue exposed to dietary AFB_1_, and previous studies have linked AFB_1_ exposure to oxidative disturbance, epithelial injury, altered mucosal integrity, and changes in intestinal microbial communities [16]. Bile acids may provide an additional link between intestinal and hepatic responses because their enterohepatic circulation is regulated by both host metabolism and microbial transformation, and bile-acid signaling can influence hepatic xenobiotic metabolism [19]. Mammalian studies have implicated microbiota–bile-acid interactions in AFB_1_ toxicity [20]; however, it remains unclear whether chronic exposure to purified AFB_1_ at feed-relevant concentrations produces broad disruption of bile-acid homeostasis in broilers or more selective, compartment-specific responses.

Accordingly, the present study used purified AFB_1_ to establish a graded exposure model in male Arbor Acres broilers over a complete 42 d production cycle. Seven dietary treatments containing nominal AFB_1_ concentrations of 0, 5, 10, 20, 50, 100, and 200 μg/kg were evaluated. The lower nominal concentrations were selected to cover a low-exposure range relevant to category-specific feed regulation, whereas the higher concentrations extended the experimental exposure range. The experiment was designed to compare biological responses across graded exposures, not to determine compliance with a single regulatory limit throughout both feeding phases. The use of a common basal diet supplemented with purified AFB_1_ minimized confounding from co-occurring mycotoxins and differences in feed composition. Growth performance and organ indices were evaluated together with hematological and serum biochemical variables, systemic and tissue redox responses, cytokines, intestinal morphology and histopathology, histological changes in major organs, and targeted bile-acid profiles across multiple anatomical compartments. The primary objective was to assess whether biological or morphological differences were detectable across the tested exposure range in the absence of statistically significant differences in growth performance.

## 2. Results

### 2.1. Dietary Exposure Was Confirmed Analytically

The concentrations of mycotoxins in samples were determined by liquid chromatography-tandem mass spectrometry (LC-MS/MS) according to our previously published method [21]. The corn lots used for diet preparation contained no detectable AFB_1_, zearalenone, deoxynivalenol, ochratoxin A, T-2 toxin, or fumonisin B_1_. AFB_1_ increased stepwise across the experimental diets, although the measured concentrations did not coincide exactly with the nominal targets in every phase. For example, the nominal 10 μg/kg diet contained 19.14 μg/kg during days 0–21 and 10.76 μg/kg during days 22–42, whereas the nominal 100 μg/kg diet contained 107.95 and 134.97 μg/kg, respectively. Nominal treatment concentrations are therefore used as group labels throughout the manuscript, with the corresponding analytical values reported in Table 1.

### 2.2. Effects of AFB_1_ on Growth Performance and Organ Indices of Broilers

Table 2 summarizes body weight at days 21 and 42 and performance over days 0–21, 22–42, and 0–42. Body weight, average daily gain (ADG), average daily feed intake (ADFI), and feed-to-gain ratio (F:G) showed no statistically significant differences for any AFB_1_ treatment (5, 10, 20, 50, 100, or 200 μg/kg; *p* > 0.05).

No statistically significant treatment differences were detected in organ indices at days 21 or 42 (Table 3). Thus, the reported analyses detected no significant differences in growth performance or gross organ indices across the tested dietary treatments.

### 2.3. Effects of AFB_1_ on Hematological Parameters of Broilers

At day 42, platelet count (PLT) and plateletcrit (PCT) differed among treatments (*p* = 0.003 and *p* = 0.004, respectively), with the highest values in the 100 and 200 μg/kg groups (Table 4). Mean platelet volume (MPV) also differed among groups (*p* = 0.004), but the pattern was non-monotonic. The remaining hematological variables did not differ significantly.

### 2.4. Serum Biochemistry, Antioxidant Measurements, and Cytokine Responses

The serum biochemical measurements listed in Table 5 showed no statistically significant response to dietary AFB_1_ concentrations of 5–200 μg/kg (*p* > 0.05).

**Figure 1:**
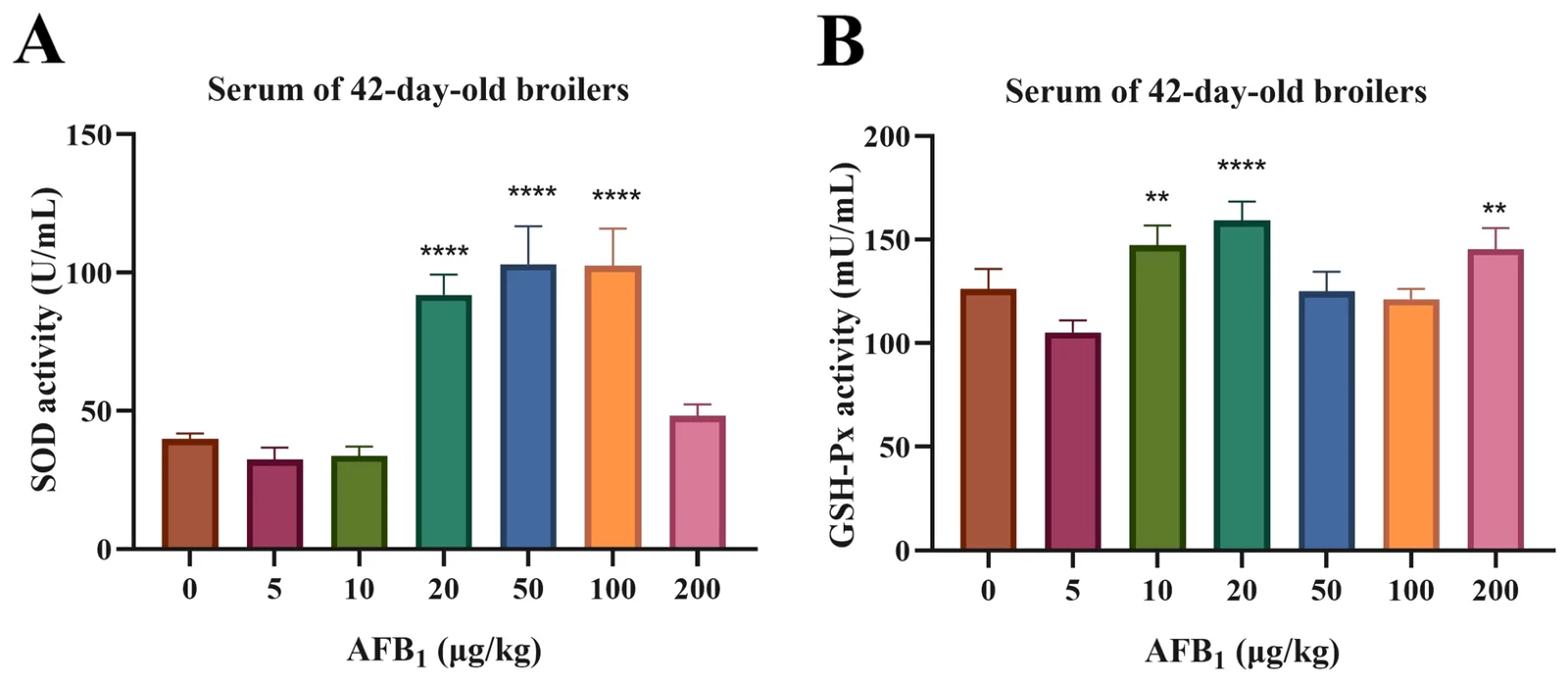
Serum antioxidant enzyme activities in AFB_1_-fed broilers: superoxide dismutase (SOD) in panel (**A**) and glutathione peroxidase (GSH-Px) in panel (**B**). Values summarize six observations per group (*n* = 6), with means and standard deviations (SD). One-way analysis of variance (ANOVA) and subsequent Duncan multiple-range comparisons were used. Asterisks mark differences from the 0 μg/kg AFB_1_ control: ** *p* < 0.01; **** *p* < 0.0001.

Figure 1 summarizes serum antioxidant enzyme activities at 42 days of age. Superoxide dismutase (SOD) activity was higher at dietary AFB_1_ concentrations of 20–100 μg/kg (*p* < 0.05), and glutathione peroxidase (GSH-Px) activity was elevated in the 10, 20, and 200 μg/kg groups (*p* < 0.05).

**Figure 2:**
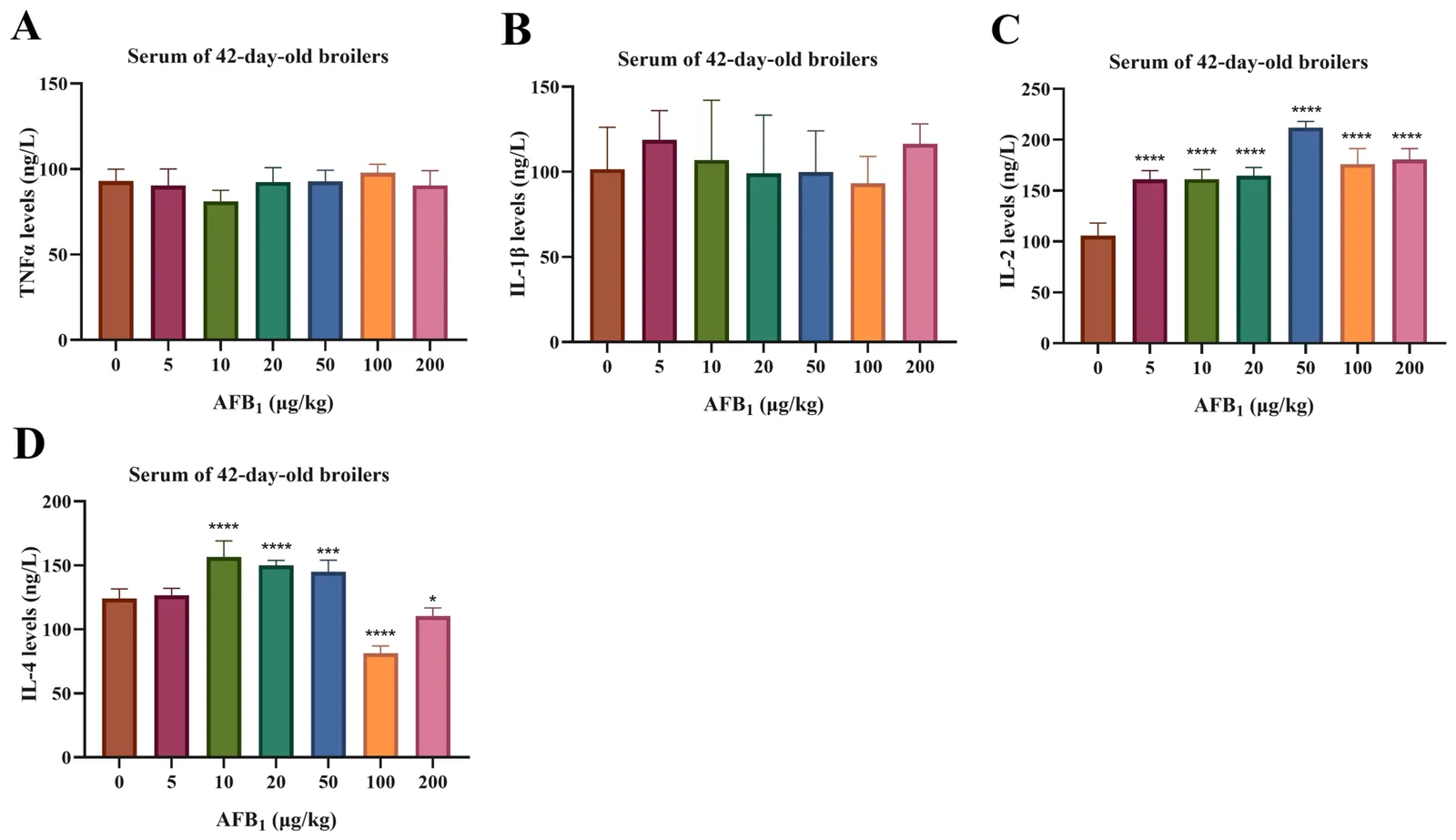
Serum concentrations of tumor necrosis factor-α (TNF-α; (**A**)), interleukin-1β (IL-1β; (**B**)), interleukin-2 (IL-2; (**C**)), and interleukin-4 (IL-4; (**D**)) across the AFB_1_ treatments. Values summarize six observations per group (*n* = 6), with means and SD. One-way ANOVA and subsequent Duncan multiple-range comparisons were used. Asterisks mark differences from the 0 μg/kg AFB_1_ control: * *p* < 0.05; *** *p* < 0.001; **** *p* < 0.0001.

The serum cytokines responded differently to AFB_1_ (Figure 2). Relative to the control, interleukin-2 (IL-2) concentrations were higher throughout the 5–200 μg/kg exposure range (*p* < 0.05). Interleukin-4 (IL-4) concentrations increased at 10–50 μg/kg but decreased at 100–200 μg/kg (*p* < 0.05). Neither tumor necrosis factor-α (TNF-α) nor interleukin-1β (IL-1β) showed a significant difference among treatments (*p* > 0.05).

### 2.5. Effects of AFB_1_ on Liver Oxidative Stress Markers

**Figure 3:**
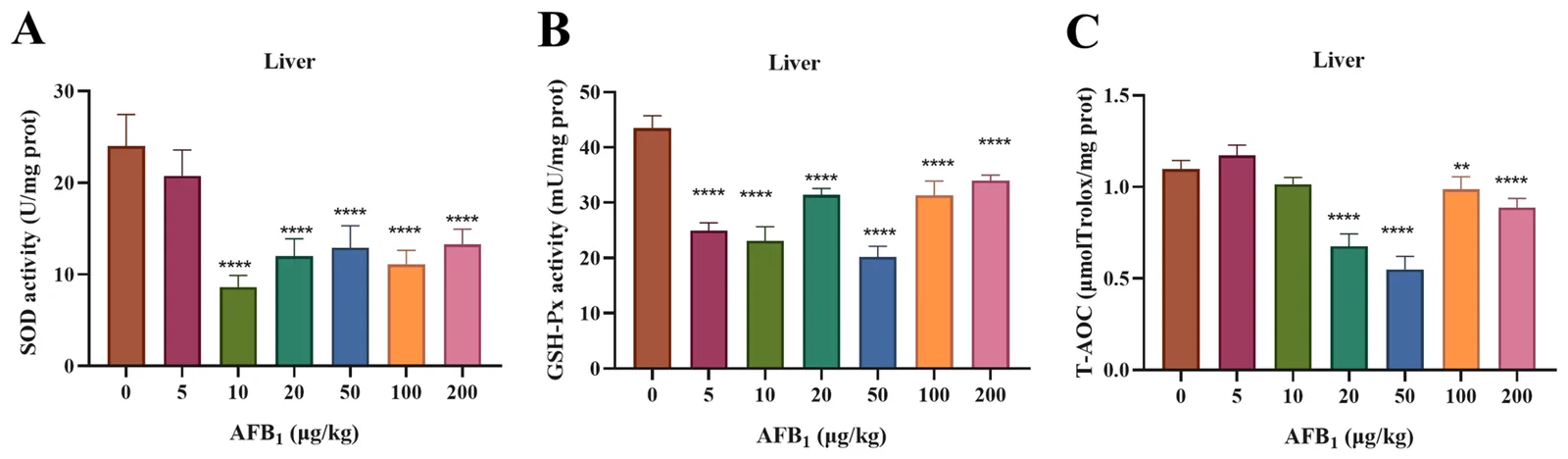
Hepatic antioxidant measurements across the AFB_1_ treatments. Panels (**A**–**C**) show SOD activity, GSH-Px activity, and total antioxidant capacity (T-AOC), respectively. Values summarize six observations per group (*n* = 6), with means and SD. One-way ANOVA and subsequent Duncan multiple-range comparisons were used. Asterisks mark differences from the 0 μg/kg AFB_1_ control: ** *p* < 0.01; **** *p* < 0.0001.

The hepatic measurements are summarized in Figure 3. SOD activity was lower at 10–200 μg/kg AFB_1_, GSH-Px activity at 5–200 μg/kg, and total antioxidant capacity (T-AOC) at 20–200 μg/kg. Each of these differences was significant relative to the control receiving 0 μg/kg AFB_1_ (*p* < 0.05).

### 2.6. Antioxidant and Cytokine Measurements in Intestinal Mucosa

**Figure 4:**
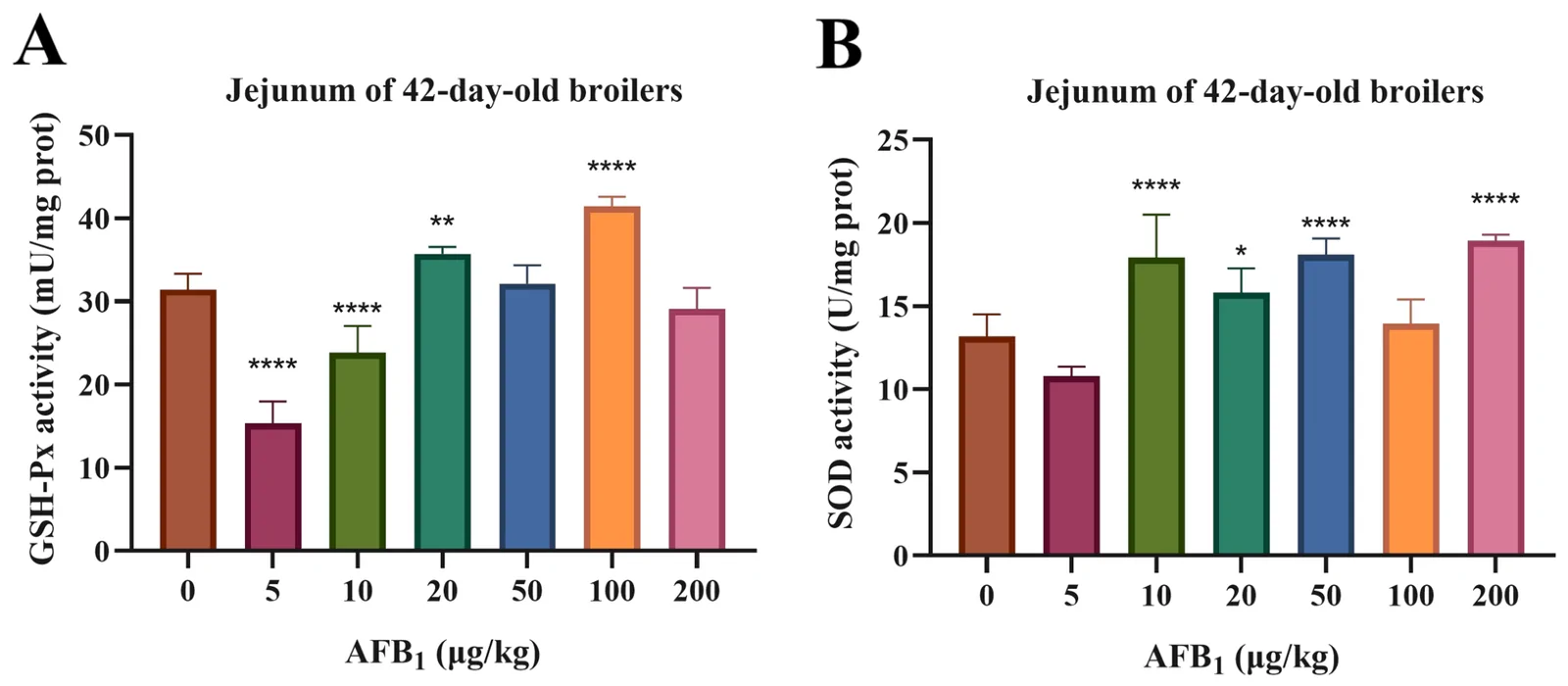
Jejunal mucosal GSH-Px (**A**) and SOD (**B**) activities across the AFB_1_ treatments. Values summarize six observations per group (*n* = 6), with means and SD. One-way ANOVA and subsequent Duncan multiple-range comparisons were used. Asterisks mark differences from the 0 μg/kg AFB_1_ control: * *p* < 0.05; ** *p* < 0.01; **** *p* < 0.0001.

Jejunal antioxidant enzyme responses varied across the exposure groups (Figure 4). GSH-Px activity was lower than the control value at 5 and 10 μg/kg AFB_1_, but higher in the high-dose groups (*p* < 0.05). SOD activity exceeded the control value at 10–50 and 200 μg/kg (*p* < 0.05).

**Figure 5:**
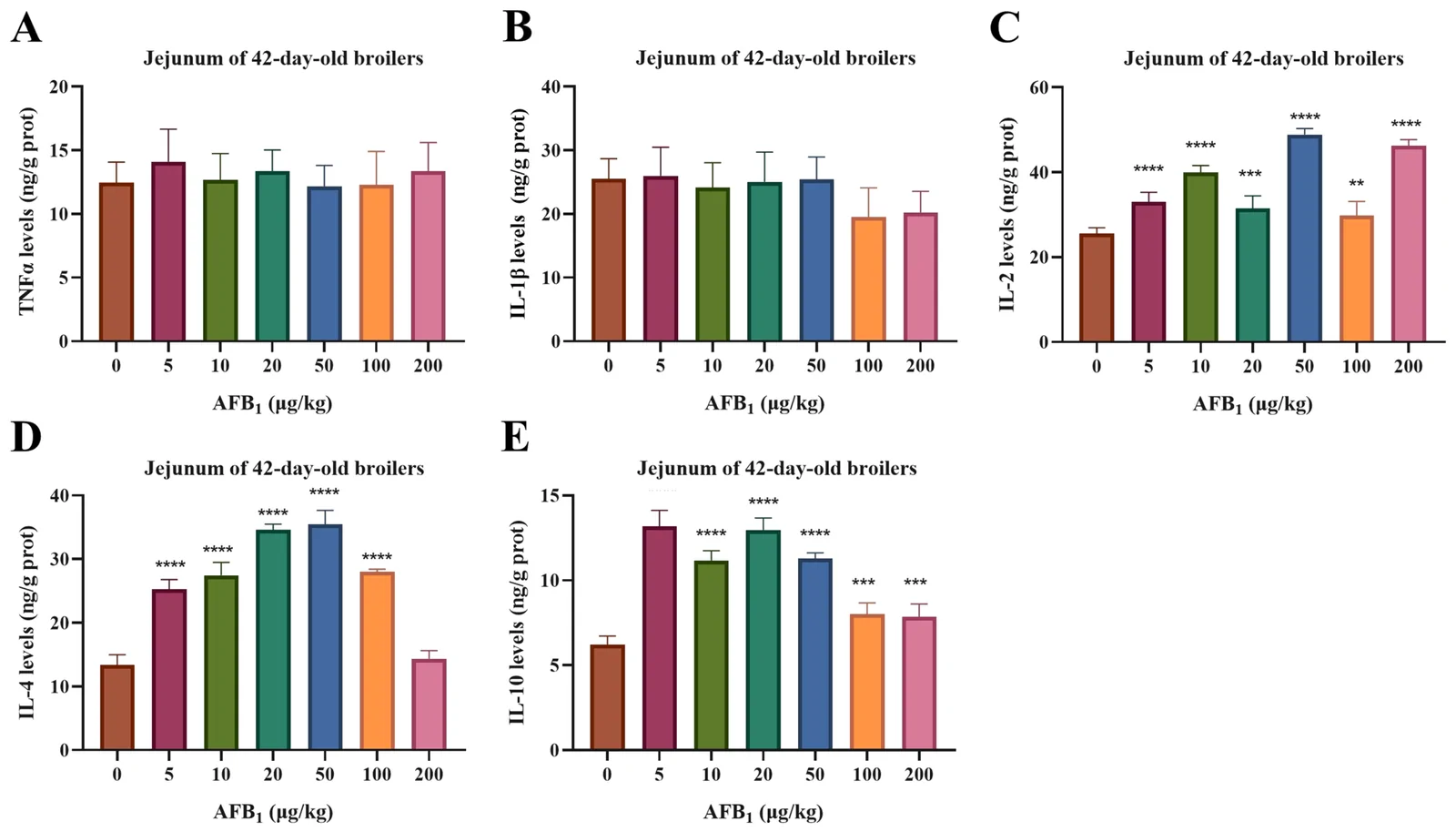
Jejunal mucosal cytokine concentrations across the AFB_1_ treatments. Panels show TNF-α (**A**), IL-1β (**B**), IL-2 (**C**), IL-4 (**D**), and interleukin-10 (IL-10; **E**). Values summarize six observations per group (*n* = 6), with means and SD. One-way ANOVA and subsequent Duncan multiple-range comparisons were used. Asterisks mark differences from the 0 μg/kg AFB_1_ control: ** *p* < 0.01; *** *p* < 0.001; **** *p* < 0.0001.

In the jejunal mucosa of 42-day-old broilers, IL-2 and interleukin-10 (IL-10) concentrations exceeded control values at 5–200 μg/kg AFB_1_, while IL-4 concentrations were higher at 5–100 μg/kg (*p* < 0.05; Figure 5). In contrast, the treatment comparisons did not identify significant differences in TNF-α or IL-1β (*p* > 0.05).

**Figure 6:**
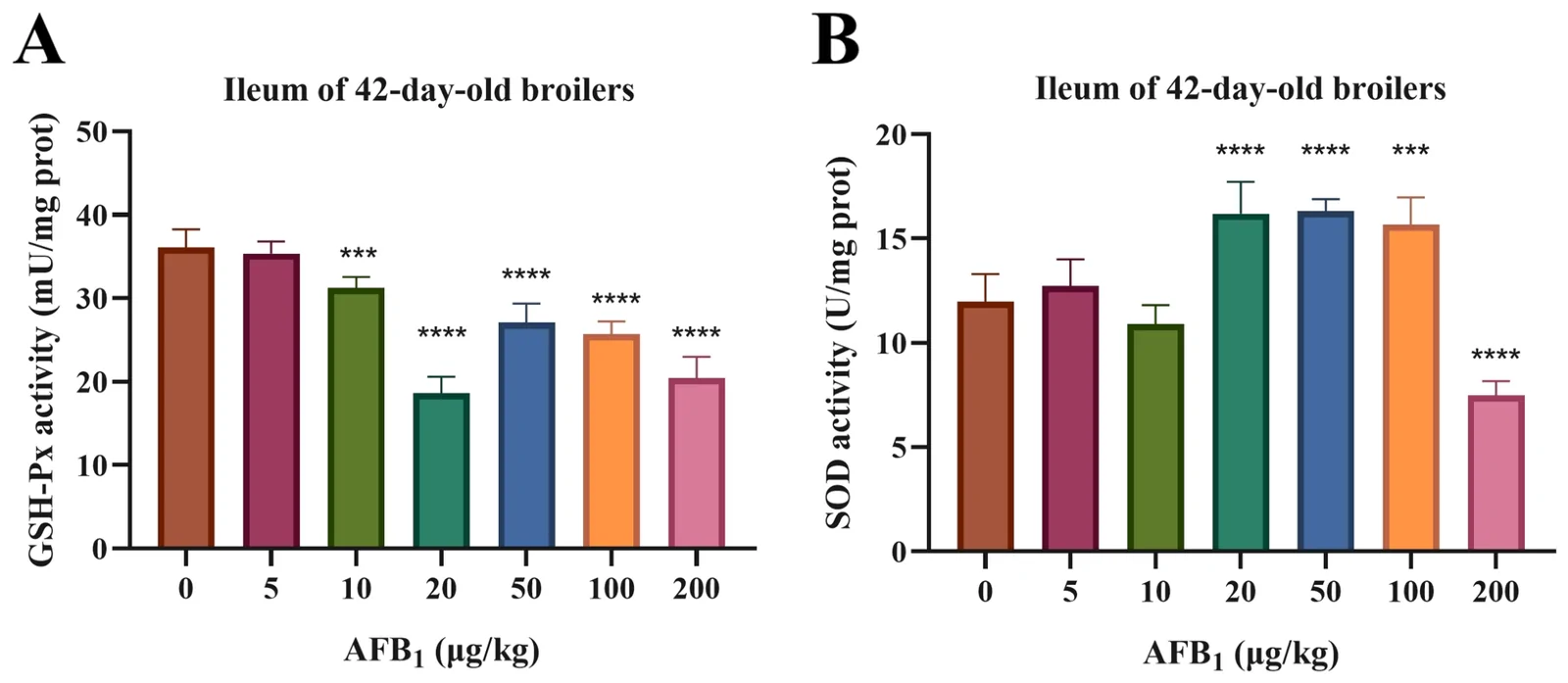
Ileal mucosal GSH-Px (**A**) and SOD (**B**) activities across the AFB_1_ treatments. Values summarize six observations per group (*n* = 6), with means and SD. One-way ANOVA and subsequent Duncan multiple-range comparisons were used. Asterisks mark differences from the 0 μg/kg AFB_1_ control: *** *p* < 0.001; **** *p* < 0.0001.

Ileal antioxidant measurements showed lower GSH-Px activity at 10–200 μg/kg AFB_1_ relative to the control (*p* < 0.05; Figure 6). SOD activity was higher at 20–100 μg/kg but lower at 200 μg/kg (*p* < 0.05), indicating that its response differed across the exposure range.

**Figure 7:**
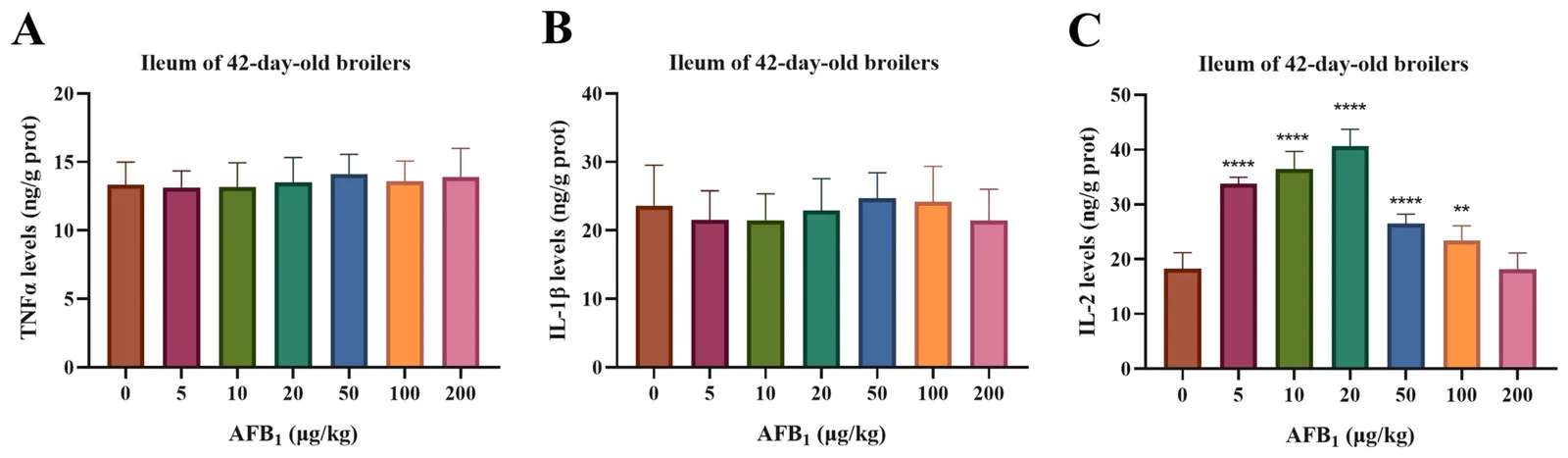
Ileal mucosal concentrations of TNF-α (**A**), IL-1β (**B**), and IL-2 (**C**) across the AFB_1_ treatments. Values summarize six observations per group (*n* = 6), with means and SD. One-way ANOVA and subsequent Duncan multiple-range comparisons were used. Asterisks mark differences from the 0 μg/kg AFB_1_ control: ** *p* < 0.01; **** *p* < 0.0001.

Figure 7 summarizes ileal cytokine measurements in 42-day-old broilers. IL-2 concentrations exceeded the control value at 5–100 μg/kg AFB_1_ (*p* < 0.05), whereas TNF-α and IL-1β did not differ significantly among treatments (*p* > 0.05).

### 2.7. Intestinal Morphology and Histopathology Analysis

Table 6 presents the jejunal and ileal morphometric measurements. Relative to the control, jejunal villus height (VH) was lower at 50–200 μg/kg AFB_1_, crypt depth (CD) was greater at 20 and 200 μg/kg, and the villus height-to-crypt depth ratio (VH:CD) was lower at 100 and 200 μg/kg (*p* < 0.05). Neither ileal VH nor CD differed significantly among treatments (overall *p* = 0.091 and *p* = 0.109, respectively). Although the overall test for ileal VH:CD was significant (*p* = 0.029), the reported post hoc comparisons identified no significant difference between an exposed group and the control. The overall result therefore reflected differences among exposed groups, rather than a consistent decrease from the control value.

**Figure 8:**
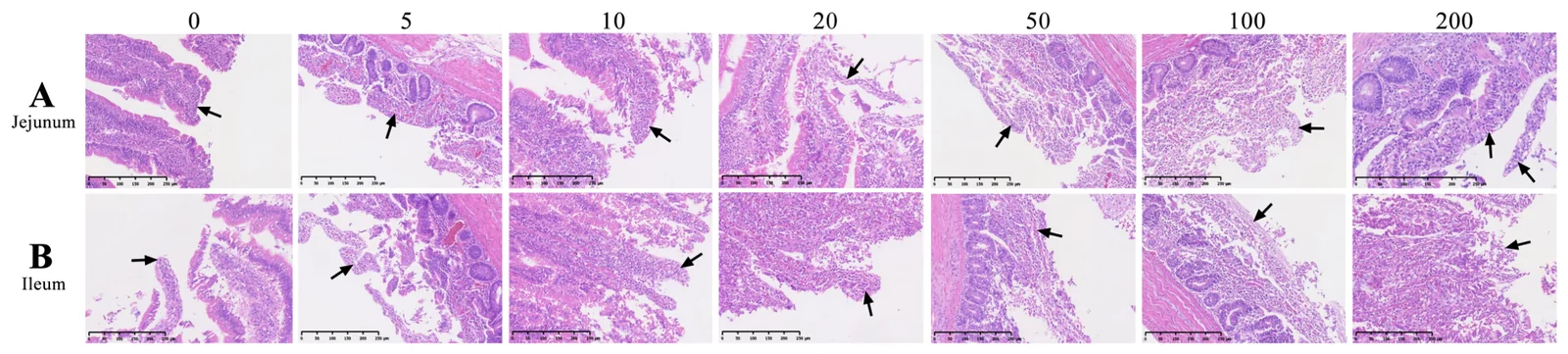
Histological appearance of the jejunum (**A**) and ileum (**B**) across the dietary AFB_1_ treatments (0, 5, 10, 20, 50, 100, and 200 μg/kg). Exfoliated mucosal epithelial cells are marked by black arrows. Sections were examined at a magnification of 100×; the scale bars correspond to 250 μm.

Figure 8 shows the intestinal histological observations. Mild lesions affecting jejunal mucosal epithelial cells were present in control birds as well as in birds receiving AFB_1_.

### 2.8. Histological Observations in Other Major Organs

**Figure 9:**
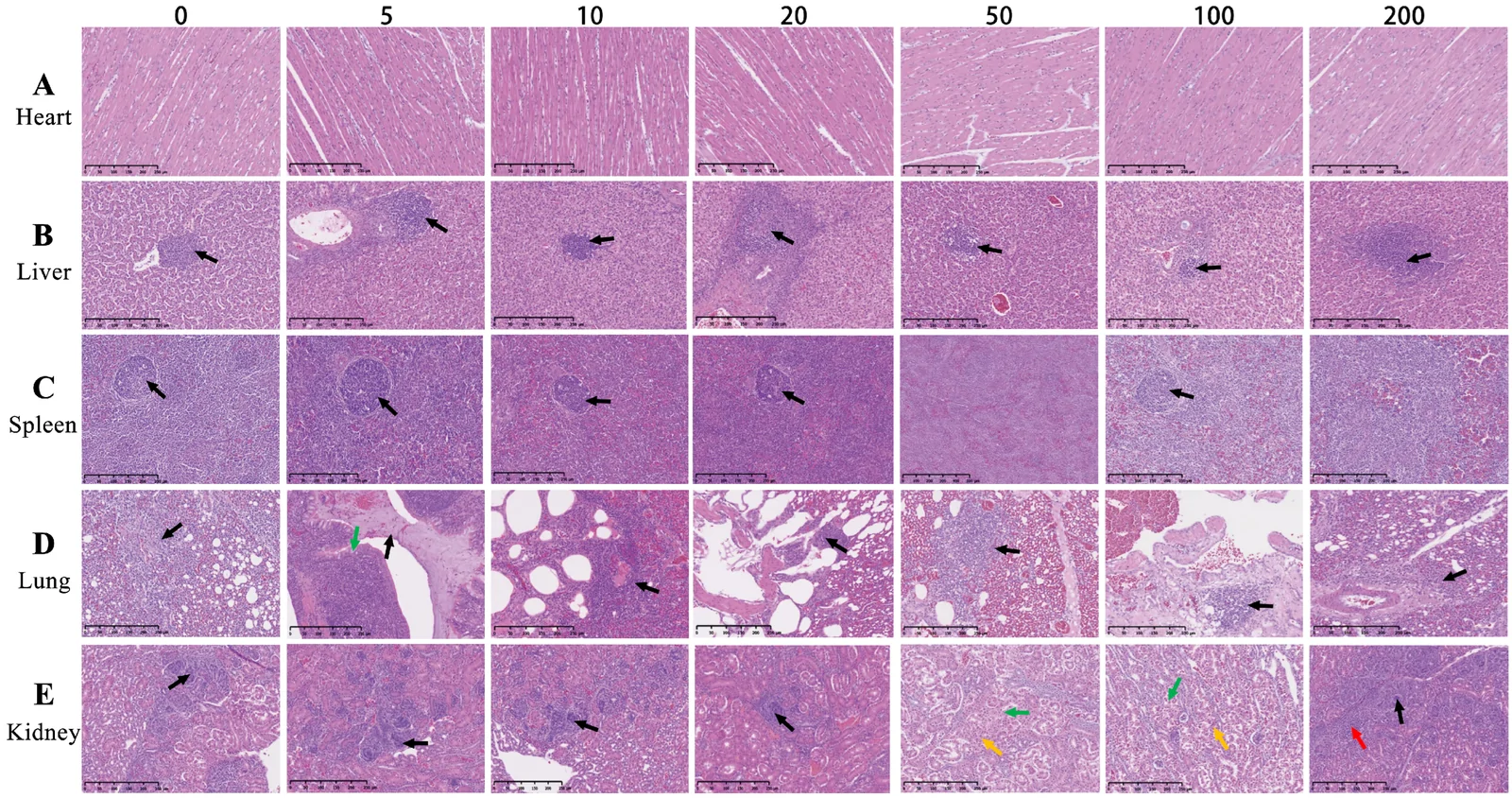
Histological appearance of the heart (**A**), liver (**B**), spleen (**C**), lung (**D**), and kidney (**E**) across the dietary AFB_1_ treatments (0, 5, 10, 20, 50, 100, and 200 μg/kg). Black arrows mark focal inflammatory-cell infiltration in the liver, germinal centers in splenic corpuscles, pulmonary inflammatory-cell infiltration, and basophilic renal tubules. Green arrows mark pulmonary secretory-cell metaplasia and renal casts. In the kidney, yellow arrows identify tubular necrosis and red arrows identify inflammatory-cell infiltration. Sections were examined at a magnification of 100×; the scale bars correspond to 250 μm.

No clear AFB_1_-related structural lesion was identified in the heart, liver, spleen, or lung (Figure 9A–D). Mild focal inflammatory-cell infiltration occurred in the liver of both control and treated birds, and occasional mild inflammatory changes were present in the lung. A mild secretory-cell metaplasia observed sporadically in the 5 μg/kg group was not reproduced at higher doses. These findings lacked a treatment-related increase in frequency or severity and were therefore considered background or incidental observations.

Renal histopathological scores showed a non-monotonic pattern of tubular injury (Figure 9E and Supplementary Table S1). No individual exposure group differed significantly from the control after adjustment. Cast formation and inflammatory infiltration occurred sporadically, whereas tubular basophilia was widespread. Other variable lesion endpoints showed no significant treatment differences after adjustment; brush border loss and tubular dilation were absent.

### 2.9. Effects of AFB_1_ on Bile Acid Profiles of Broilers

**Figure 10:**
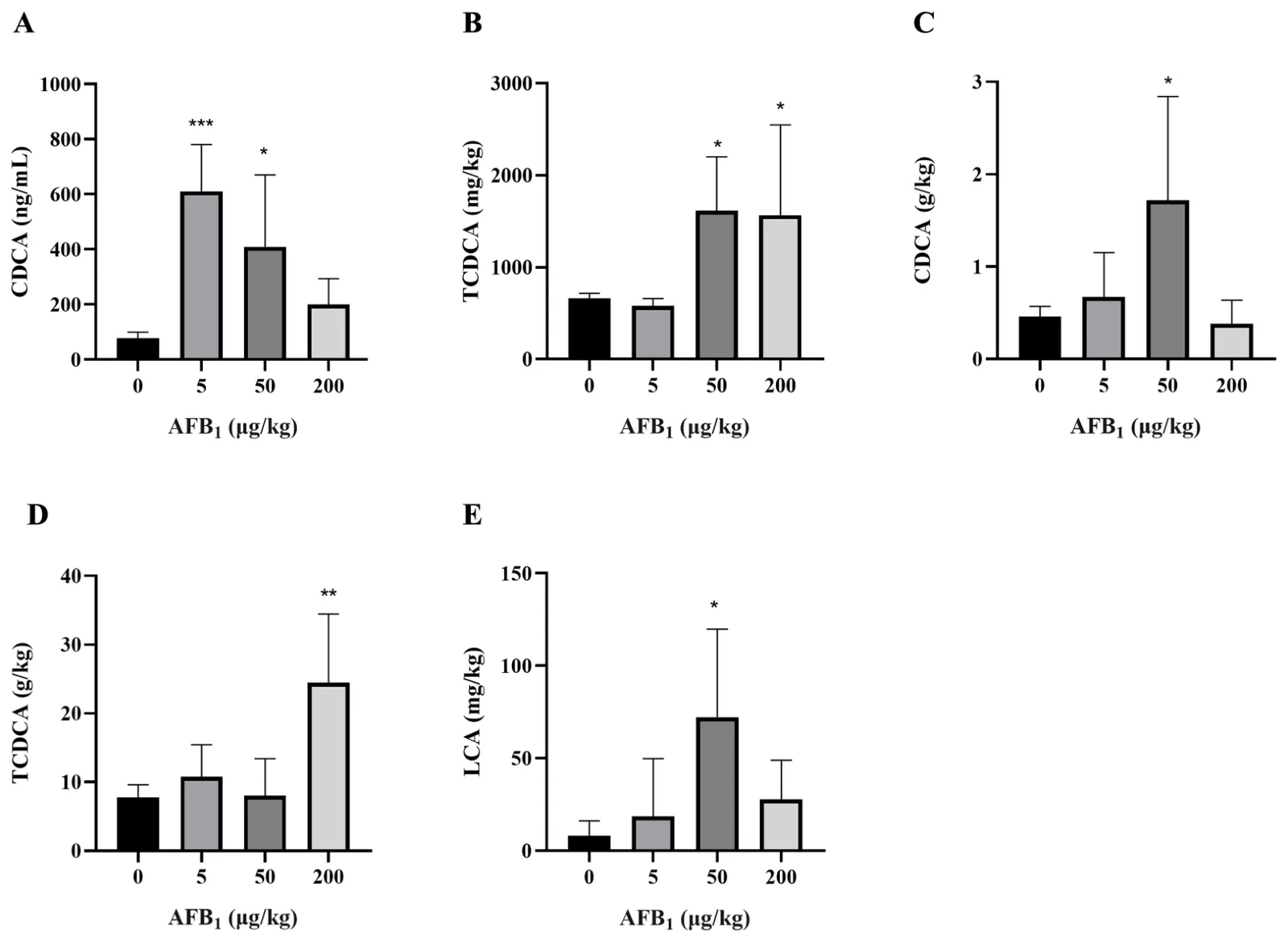
Selected bile-acid measurements in broilers receiving 0, 5, 50, or 200 μg/kg nominal dietary AFB_1_. Panels show serum chenodeoxycholic acid (CDCA; (**A**)), hepatic taurochenodeoxycholic acid (TCDCA; (**B**)), CDCA in duodenal contents (**C**), TCDCA in jejunal contents (**D**), and lithocholic acid (LCA) in colorectal contents (**E**). Means and SD summarize measurements from six birds per group (*n* = 6), each representing a different replicate cage. One-way analysis of variance (ANOVA) and subsequent Duncan multiple-range comparisons were used. Asterisks mark differences from the 0 μg/kg AFB_1_ control: * *p* < 0.05; ** *p* < 0.01; *** *p* < 0.001.

Among the four treatment groups included in bile-acid profiling (0, 5, 50, and 200 μg/kg), the reported analyses identified differences in selected bile acids in specific biological compartments. In serum, chenodeoxycholic acid (CDCA) was significantly elevated at 5 and 50 μg/kg AFB_1_ (Figure 10A). Hepatic taurochenodeoxycholic acid (TCDCA) levels increased significantly under 50 and 200 μg/kg AFB_1_ exposure (Figure 10B). For intestinal bile acids, duodenal CDCA increased at 50 μg/kg, jejunal TCDCA increased at 200 μg/kg, and colorectal lithocholic acid (LCA) was markedly upregulated at 50 μg/kg (Figure 10C–E). No statistically significant differences were reported for ileal bile-acid profiles or the other measured biliary bile acids among the four analyzed treatment groups (Figure S1).

## 3. Discussion

Under the conditions of this 42 d experiment, the reported analyses did not detect significant treatment differences in growth performance or major organ indices, whereas selected biochemical measurements and jejunal morphology differed among groups. These observations suggest that growth measurements alone may not fully describe the biological response to dietary AFB_1_ exposure. They do not establish equivalence of growth across treatments or demonstrate that every biomarker difference represents an adverse effect. The reduction in jejunal villus height across the higher-dose groups represents a more consistent descriptive pattern than isolated or non-monotonic biomarker differences, which require confirmation after accounting for multiplicity.

The lack of statistically significant growth differences at nominal concentrations up to 200 μg/kg is consistent with some previous low-dose broiler studies but contrasts with reports of reduced feed intake or growth at comparable or higher exposures [17,22,23,24]. Differences in the exposure model may partly explain these inconsistencies. Naturally contaminated corn or fungal-cultured ingredients may contain additional mycotoxins, fungal metabolites, or altered nutrient profiles, making it difficult to attribute observed effects exclusively to AFB_1_ [25]. In contrast, the present study used purified AFB_1_ added to a common nutritionally matched basal diet, thereby minimizing these confounding factors and improving attribution of the observed responses to AFB_1_ itself. This design is particularly useful for dose–response and hazard characterization, although it does not reproduce all aspects of naturally contaminated feed.

Interpretation of the lower exposure groups requires particular caution because nominal and measured AFB_1_ concentrations differed. During days 0–21, the nominal 10 μg/kg diet contained 19.14 μg/kg, approximately 1.9 times its target concentration and close to the 20.96 μg/kg measured in the nominal 20 μg/kg diet. These treatments therefore provided poorly separated dietary concentrations during the starter phase. During days 22–42, the corresponding concentrations were 10.76 and 19.78 μg/kg. Consequently, responses in the nominal 10 μg/kg group cannot establish an effect threshold at an actual dietary concentration of 10 μg/kg, and the two treatment labels should not be interpreted as well-separated exposures throughout the experiment. Nominal concentrations are retained for group identification, whereas interpretation of exposure-related responses must consider the measured concentrations in both feeding phases.

PLT and PCT were higher at selected exposures, whereas the reported analyses detected no significant differences in most other hematological variables or routine serum biochemical indices. Because PCT depends partly on cell count and volume, PLT and PCT should not be interpreted as independent evidence of toxicity. Avian thrombocytes can participate in innate immune responses [26]. Altered production, release, or peripheral turnover could contribute to the observed count-related differences, but bone marrow responses, thrombocyte activation, and vascular inflammation were not assessed. These mechanisms therefore remain speculative, and the present measurements do not establish a hypercoagulable state. Similarly, increases in serum SOD and GSH-Px activities may be compatible with adaptive or compensatory responses, but neither an adaptive mechanism nor improved antioxidant function was established.

In contrast, hepatic antioxidant responses showed a more consistent pattern across the dose range. Hepatic SOD activity was reduced in the nominal 10–200 μg/kg groups, GSH-Px activity in the nominal 5–200 μg/kg groups, and T-AOC in the nominal 20–200 μg/kg groups. These findings should be interpreted in light of the differences between nominal and measured dietary AFB_1_ concentrations. Oxidative disturbance is a well-established component of AFB_1_ toxicity in poultry [6,8], particularly in the liver, which is a major site of AFB_1_ bioactivation. However, no statistically significant treatment differences were detected in serum ALT, AST, total bilirubin, or the liver index, and qualitative examination of hepatic sections stained with hematoxylin and eosin (H&E) did not identify clear treatment-related lesions. Thus, altered hepatic antioxidant status was observed without clear accompanying biochemical or histological evidence of liver injury at day 42. Because lipid peroxidation was not assessed, the decreases in hepatic antioxidant enzyme activities cannot by themselves establish oxidative tissue injury. They indicate altered antioxidant status, while their relation to oxidative damage remains unresolved. Moreover, measurements obtained at a single sampling time cannot establish whether antioxidant changes preceded tissue injury. Future studies incorporating measurements of lipid peroxidation, the balance between reduced glutathione (GSH) and oxidized glutathione (GSSG), oxidative DNA damage, and quantitative histopathology, together with sampling at multiple time points, would help clarify the biological significance and temporal progression of these changes.

The cytokine profile similarly indicated immune modulation rather than generalized inflammation. TNF-α and IL-1β remained unchanged in serum and intestinal mucosa, whereas IL-2, IL-4, and IL-10 exhibited selective and frequently non-monotonic responses. Previous studies have shown that AFB_1_ can modulate immune function in poultry [27,28], but the present results do not support a simple pro-inflammatory phenotype. Nor do they provide sufficient evidence to assign a specific T helper 1 (Th1)/T helper 2 (Th2) shift or to define overt immunotoxicity. These selective cytokine differences may indicate altered immune signaling, but their functional consequences and any compensatory role remain unresolved.

The intestinal findings support an effect on morphology more directly than they establish epithelial injury. The reported analyses showed lower jejunal villus height in the nominal 50–200 μg/kg groups, whereas intestinal antioxidant responses were variable and often non-monotonic. Previous studies have reported AFB_1_-associated epithelial and barrier alterations [29,30], but those findings do not substitute for direct evidence in the present experiment. Mild epithelial alterations occurred in both control and treated birds, and their incidence and severity were not quantified. Accordingly, the present intestinal conclusion is restricted to the observed reduction in jejunal villus height; increased epithelial injury or impaired barrier function was not established.

The renal findings require cautious interpretation. Semiquantitative scoring by an assessor unaware of treatment dose showed higher median tubular necrosis scores at 50 and 100 μg/kg and a significant overall difference in severity across treatments. However, no individual exposure group differed significantly from the control after adjustment of the exact pairwise comparisons, and none of the incidence comparisons with the control was significant after Holm adjustment, despite a significant overall incidence difference. The absence of recorded tubular necrosis at 200 μg/kg, together with necrosis scores in the 5 μg/kg group, indicates a non-monotonic pattern rather than a straightforward dose–severity relationship. Although the kidney is a recognized extrahepatic target of AFB_1_ [31,32], these exploratory findings were based on a limited number of samples and assessment by a single observer. Larger samples, reproducibility assessment across observers, and avian-relevant renal functional measurements are needed before these lesions can be considered a robust critical endpoint for dose–response or regulatory assessment.

The reported bile-acid differences were selective and compartment-specific within the four analyzed treatment groups; these measurements did not characterize the entire enterohepatic bile-acid pool. Serum CDCA increased at 5 and 50 μg/kg, hepatic TCDCA at 50 and 200 μg/kg, jejunal TCDCA at 200 μg/kg, and colorectal LCA at 50 μg/kg, whereas no statistically significant differences in biliary taurocholic acid (TCA) or TCDCA were reported among the analyzed groups. AFB_1_ has been associated with altered bile-acid signaling and microbiota-dependent bile-acid metabolism in mammalian models [20,33], and bile acids may influence hepatic AFB_1_ biotransformation in broilers [34]. However, the present findings do not support overt cholestasis or a uniform expansion of the bile-acid pool. Instead, they suggest selective changes in the handling or transformation of individual bile-acid species at specific anatomical sites.

These bile-acid responses provide mechanistic hypotheses but do not establish causality. Increased duodenal CDCA could arise from changes in bile-acid deconjugation, intestinal absorption, or enterohepatic exchange [19], whereas the increase in jejunal TCDCA at 200 μg/kg is compatible with reduced microbial deconjugation because bacterial bile salt hydrolases catalyze conversion of conjugated to unconjugated bile acids. Such spatial distribution characteristics are consistent with the mechanism reported by Mao et al. [20], in which AFB_1_ inhibits bile salt hydrolase (BSH) activity in the ileum of piglets. However, BSH activity or gene abundance, bile-acid transporters, cholesterol 7α-hydroxylase (CYP7A1), and farnesoid X receptor (FXR)-related signaling were not determined in this study.

From a feed-safety perspective, the findings support evaluating growth, biochemical measurements, and tissue morphology together [35]. The reported analyses detected no significant growth differences, while jejunal villus height was lower in several higher-exposure groups and selected biochemical endpoints differed at other exposures. These observations do not establish a formal ranking of endpoint sensitivity or a progression from early biological responses to later tissue injury. In particular, they do not demonstrate that 20 μg/kg is a safe dietary limit or that 10 μg/kg is unsafe. Interpretation must account for differences between nominal and measured concentrations, the overlap of the nominal 10 and 20 μg/kg groups during the starter phase, multiple testing, and non-monotonic responses. The results identify outcomes for further investigation rather than a regulatory threshold.

Exposure duration should also be considered when translating these findings to feed-safety standards. The 42 d exposure period represented the complete commercial grow-out cycle for the fast-growing Arbor Acres broilers used in this study. However, the results should not be extrapolated directly to slower-growing broilers maintained for substantially longer production periods. The biological consequences of a given dietary concentration depend not only on feed concentration but also on duration of exposure, age, feed intake, and physiological sensitivity. A concentration associated primarily with early biological responses during a 42 d production cycle might produce different outcomes during more prolonged continuous exposure. Future standard-oriented studies should therefore evaluate AFB_1_ concentration together with exposure duration and production stage, particularly when applying safety limits across broiler production systems with markedly different grow-out periods.

Several limitations should be considered. Growth performance was evaluated using six replicate cages per treatment, and blood and biochemical measurements were based on one bird from each cage. Additional renal histopathological scoring was performed in a subset of birds from different replicate cages. These sample sizes limit precision and may reduce the ability to detect modest effects, particularly for variable immune and metabolic endpoints. Non-significant results do not establish equivalence or exclude biological effects. Bile-acid profiling was restricted to four treatment groups, and the variability in duodenal CDCA and colorectal LCA further limits dose–response interpretation. Measurements of bile salt hydrolase activity, transporters, synthetic enzymes, and receptor signaling were not available to establish mechanisms. Renal scoring was performed by a single assessor, without evaluation of interobserver reproducibility, and intestinal epithelial alterations were not quantified. Finally, the reported analyses of continuous outcomes relied on one-way ANOVA followed by Duncan’s multiple-range test without a study-wide multiplicity correction. Holm correction was applied to the additional renal analyses, but this does not address multiplicity across the other endpoints. Formal linear or quadratic trend analyses and benchmark-dose modeling were not performed, so the categorical comparisons should not be interpreted as a validated quantitative exposure–response relationship. Isolated or non-monotonic biomarker differences remain exploratory. The findings support further evaluation of intestinal morphology and selected biological endpoints alongside growth performance, rather than a definitive ranking of endpoint sensitivity or a new dietary safety threshold.

## 4. Conclusions

Under the conditions of this 42 d experiment, the reported analyses detected no significant differences in broiler growth performance or major organ indices across nominal dietary AFB_1_ concentrations of 0–200 μg/kg. Jejunal villus height was lower in the nominal 50–200 μg/kg groups, while selected antioxidant, cytokine, hematological, and bile-acid measurements showed variable responses. Additional renal scoring identified overall differences in tubular necrosis severity and incidence, but no individual exposure group differed significantly from the control after adjustment of exact comparisons. These observations suggest that selected biological and morphological responses may occur without detectable growth impairment. However, the small sample sizes, multiple comparisons, non-monotonic findings, and differences between nominal and measured exposures limit causal and quantitative dose–response interpretation. The results do not establish a dietary safety threshold or a formal ranking of endpoint sensitivity. Larger studies with appropriate multiplicity control and reproducible functional or tissue endpoints are needed to assess the robustness and practical significance of these findings.

## 5. Materials and Methods

### 5.1. AFB_1_ Standard, Diet Preparation, and Experimental Design

AFB_1_ standard (purity ≥ 99%) was obtained from Shandong Meizheng Bio-Tech Co., Ltd. (Rizhao, China). Methanol and acetonitrile were chromatographic grade (Thermo Fisher Scientific, Waltham, MA, USA), while dimethyl sulfoxide (DMSO) and anhydrous ethanol were of analytical grade. A corn–soybean meal basal diet was prepared for starter (days 0–21) and grower (days 22–42) phases. The formulation and nutritional composition of the basal diet were identical to those reported in our previous study [36]. The diets were formulated according to National Research Council (NRC, 1994) recommendations [37].

For each AFB_1_ treatment, the required amount of standard was dissolved in 10 mL DMSO, mixed thoroughly with premix, and then incorporated into the complete feed by stepwise dilution. Seven dietary treatments were prepared with nominal AFB_1_ concentrations of 0, 5, 10, 20, 50, 100, and 200 μg/kg. All diets had the same formulation within each feeding phase and differed only in AFB_1_ supplementation.

One-day-old male Arbor Acres broiler chicks were assigned to the seven dietary treatments, with six replicate cages per treatment and six birds per cage. The feeding trial lasted 42 d. Birds were housed in three-tier cages in the Animal Digestion and Metabolism Laboratory, College of Animal Science and Technology, China Agricultural University, and received mash feed and water ad libitum. Animal procedures were approved by the China Agricultural University Laboratory Animal Welfare and Animal Experimental Ethical Inspection Committee (approval no. AW91903202-1-3).

### 5.2. Sample Collection

On days 21 and 42, birds were fasted for 12 h and six birds per treatment were selected for sampling. Blood was collected from the wing vein. After standing for 30 min, samples were centrifuged at 3000 rpm for 10 min to obtain serum, which was stored at −20 °C. After blood collection, the broilers were euthanized via exsanguination through the jugular vein. Liver and mucosal scrapings from the duodenum, jejunum, and ileum were snap-frozen in liquid nitrogen and stored frozen for biochemical analysis. Heart, liver, spleen, lung, kidney, jejunum, and ileum were fixed in 4% paraformaldehyde. Bile and contents from the duodenum, jejunum, ileum, and colorectal segment were collected for bile-acid analysis and stored at −80 °C. Additional renal histopathological scoring was performed in a subset of sampled birds from different replicate cages.

### 5.3. Growth Performance and Organ Indices

Body weight was recorded at the beginning of the experiment and after fasting on days 21 and 42. Residual feed was weighed by replicate cage at the same time points. ADG, ADFI, and F:G were calculated for days 0–21, 22–42, and 0–42. At necropsy, heart, liver, spleen, lung, and kidney were weighed. Organ indices were calculated as organ weight (g)/live body weight (kg).

### 5.4. Hematology, Serum Biochemistry, Redox Status, and Cytokines

Hematological variables were measured using a TEK-II automated hematology analyzer and included WBC, RBC, HGB, HCT, MCV, MCH, MCHC, PLT, PCT, MPV, and PDW. ALT, AST, ALP, TP, ALB, TG, GLU, UREA, TBIL, CREA, IgA, IgG, and IgM were measured using a Mindray BS-420 automated biochemical analyzer (Mindray Bio-Medical Electronics Co., Ltd., Shenzhen, China).

For tissue assays, 0.2 g of tissue was homogenized in nine volumes of physiological saline to produce a 10% homogenate. Protein concentration was determined using a bicinchoninic acid (BCA) assay (Beyotime, Shanghai, China). SOD, GSH-Px, T-AOC, TNF-α, IL-1β, IL-2, IL-4, and IL-10 were measured in serum or tissue homogenates using commercial assay kits from Shanghai Hengyuan Biological Technology Co., Ltd. (Shanghai, China), following the manufacturers’ instructions. Tissue measurements were normalized to protein concentration where appropriate.

### 5.5. Histopathology and Intestinal Morphometry

Fixed tissues were held in 4% paraformaldehyde for 48 h, trimmed to blocks no larger than 1.5 cm × 1.5 cm and 0.3 cm thick, washed in running water, dehydrated through graded ethanol, cleared with xylene, and embedded in paraffin. Sections were cut at 4 μm, mounted on glass slides, dried at 64 °C for 2 h, and stained with hematoxylin and eosin. Whole-slide images were obtained with a Hamamatsu NanoZoomer S60 scanner (Hamamatsu Photonics K.K., Hamamatsu, Japan). Tissue morphology was evaluated from the scanned sections. Jejunal and ileal villus height and crypt depth were measured, and the villus height-to-crypt depth ratio was calculated. Renal histopathology was additionally evaluated using semiquantitative scoring in birds selected from different replicate cages. A single assessor who was unaware of the treatment dose evaluated renal tubular necrosis, cast formation, brush border loss, tubular dilation, tubular basophilia, and inflammatory cell infiltration. Each lesion was assigned a composite score for each bird based on an overall assessment of the evaluated microscopic fields, using the following ordinal scale: 0, no lesion; 1, minimal; 2, mild; 3, moderate; and 4, marked.

### 5.6. Targeted Bile-Acid Analysis

The nominal 5, 50, and 200 μg/kg groups were chosen to represent low, intermediate, and high exposure levels within the experimental concentration range, respectively. Six birds per group, one from each replicate cage, were included in the analysis. Samples from the nominal 10, 20, and 100 μg/kg groups were not analyzed for bile acids. Liver and intestinal-content samples were freeze-dried before bile-acid analysis. Bile acids were quantified in serum, bile, liver, and duodenal, jejunal, ileal, and colorectal contents using a targeted LC–MS/MS method previously established in our laboratory. The panel included taurine-conjugated, glycine-conjugated, and unconjugated bile acids, including TCDCA, TCA, taurohyodeoxycholic acid (THDCA), taurohyocholic acid (THCA), taurolithocholic acid (TLCA), tauroursodeoxycholic acid (TUDCA), taurodeoxycholic acid (TDCA), glycohyodeoxycholic acid (GHDCA), glycochenodeoxycholic acid (GCDCA), glycohyocholic acid (GHCA), glycolithocholic acid (GLCA), glycocholic acid (GCA), glycoursodeoxycholic acid (GUDCA), glycodeoxycholic acid (GDCA), hyodeoxycholic acid (HDCA), CDCA, hyocholic acid (HCA), LCA, ursodeoxycholic acid (UDCA), cholic acid (CA), deoxycholic acid (DCA), and muricholic-acid species. Results were evaluated for individual bile acids in each biological compartment.

### 5.7. Statistical Analysis

Renal histopathological scores were analyzed separately from the continuous outcomes. Renal lesion severity was analyzed using permutation-based Kruskal–Wallis and Fisher–Freeman–Halton exact tests, respectively, with Holm adjustment for multiple comparisons. Continuous outcomes were analyzed by one-way analysis of variance in SPSS (v21.0), followed by Duncan’s multiple-range test for post hoc comparisons. *p* < 0.05 was considered statistically significant. The replicate cage was the experimental unit for growth-performance data. For the reported blood, biochemical, and intestinal morphology measurements, one bird per replicate cage was used (*n* = 6 per treatment, where indicated). Continuous data are presented as means with SEM in tables and as mean ± SD in figures. Nominal dietary AFB_1_ concentrations are used as treatment labels, while analytically measured concentrations are reported in Table 1. No study-wide multiplicity correction was applied to the continuous outcomes.

## Figures and Tables

**Table 1:** Nominal and measured AFB_1_ concentrations in the experimental diets.

Nominal AFB_1_ (μg/kg)	Expected Value (μg/kg)	Measured 0–21 d (μg/kg)	Measured 22–42 d (μg/kg)
0	0	0.28	0.13
5	5	7.58	5.43
10	10	19.14	10.76
20	20	20.96	19.78
50	50	42.48	55.92
100	100	107.95	134.97
200	200	205.82	205.75

Note: Nominal AFB_1_, intended spiked concentration of experimental diets. Measured values are analytically determined AFB_1_ concentrations (μg/kg) for two experimental phases (0–21 d and 22–42 d). Nominal concentrations identify treatment groups and do not represent the exact concentrations consumed throughout the experiment. The nominal 10 and 20 μg/kg groups had closely overlapping measured dietary concentrations during days 0–21.

**Table 2:** Broiler growth measurements across the dietary AFB_1_ treatments.

Items	Concentration of AFB_1_ (μg/kg)							SEM	*p*-Values
	0	5	10	20	50	100	200		
Initial BW (g)	48.75	48.76	48.51	48.53	50.25	48.47	47.36	0.35	0.090
21 d BW (kg)	0.55	0.54	0.50	0.52	0.52	0.51	0.49	0.02	0.134
42 d BW (kg)	2.09	2.00	1.95	1.97	1.97	2.06	1.97	0.08	0.849
0–21 d									
ADG (g)	24.85	24.29	22.74	23.56	23.52	22.78	21.98	0.76	0.159
ADFI (g)	36.12	34.58	35.47	35.16	37.29	33.74	34.67	0.94	0.200
F:G	1.46	1.42	1.56	1.50	1.60	1.48	1.58	0.05	0.109
22–42 d									
ADG (g)	73.54	69.16	68.48	68.71	69.13	73.32	70.16	3.14	0.823
ADFI (g)	120.64	117.53	112.86	117.28	116.29	119.37	106.19	5.11	0.498
F:G	1.64	1.70	1.65	1.71	1.68	1.63	1.55	0.06	0.620
0–42 d									
ADG (g)	49.79	47.28	46.17	46.69	46.88	48.67	46.66	1.85	0.808
ADFI (g)	78.38	76.06	74.16	76.22	76.79	76.56	70.43	2.72	0.513
F:G	1.58	1.61	1.61	1.63	1.64	1.57	1.53	0.04	0.642

Note: Six replicates were evaluated (*n* = 6). SEM denotes the standard error of the mean. Abbreviations for growth measurements are BW (body weight), ADG (average daily gain), ADFI (average daily feed intake), and F:G (feed-to-gain ratio).

**Table 3:** Organ indices of broilers receiving the different AFB_1_ diets.

Items	Concentration of AFB_1_ (μg/kg)							SEM	*p*-Values
	0	5	10	20	50	100	200		
21 d									
Heart (g/kg)	7.61	7.54	7.25	7.42	7.38	7.88	6.83	0.34	0.498
Liver (g/kg)	23.33	26.27	24.19	22.98	26.14	24.40	24.59	1.77	0.796
Spleen (g/kg)	0.92	0.82	0.97	0.77	0.87	0.76	0.96	0.08	0.332
Lung (g/kg)	8.07	8.35	9.38	7.20	8.85	7.14	9.35	0.66	0.098
Kidney (g/kg)	7.84	8.06	8.09	7.09	9.04	7.51	9.12	0.54	0.118
42 d									
Heart (g/kg)	4.88	4.27	4.36	4.77	4.35	4.26	4.41	0.23	0.366
Liver (g/kg)	19.20	19.84	18.51	21.38	21.12	19.51	18.06	0.78	0.052
Spleen (g/kg)	1.30	1.13	1.22	1.23	1.26	1.18	1.71	0.21	0.568
Lung (g/kg)	6.43	5.60	5.37	5.88	6.50	5.82	5.89	0.50	0.695
Kidney (g/kg)	6.36	5.74	5.97	6.53	6.28	5.92	5.95	0.28	0.507

Note: Results are based on six replicates (*n* = 6); SEM denotes the standard error of the mean.

**Table 4:** Hematological measurements in broilers receiving the different AFB_1_ diets.

Items	Concentration of AFB_1_ (μg/kg)							SEM	*p*-Values
	0	5	10	20	50	100	200		
WBC (10^9^/L)	119.23	118.67	114.34	119.98	120.72	121.85	118.86	1.59	0.062
RBC (10^12^/L)	2.68	2.73	2.77	2.73	3.13	2.93	2.94	0.12	0.128
HGB (g/L)	60.50	62.33	58.00	54.17	74.83	56.13	68.67	7.65	0.498
HCT (L/L)	0.26	0.28	0.28	0.27	0.31	0.29	0.29	0.01	0.112
MCV (fL)	95.23	101.77	100.12	98.48	83.87	98.35	99.07	5.67	0.371
MCH (pg)	21.15	22.63	20.67	19.65	23.58	20.93	22.90	1.37	0.415
MCHC (g/L)	206.50	211.00	195.67	189.33	227.00	202.17	218.83	12.52	0.393
PLT (10^9^/L)	15.40 ^c^	20.75 ^bc^	14.00 ^c^	15.00 ^c^	26.00 ^abc^	40.83 ^a^	37.00 ^ab^	5.10	0.003
PCT (L/L)	0.02 ^c^	0.02 ^bc^	0.02 ^c^	0.02 ^c^	0.03 ^abc^	0.04 ^a^	0.04 ^ab^	0.01	0.004
MPV (fL)	10.86 ^b^	11.40 ^a^	11.32 ^a^	11.25 ^a^	11.40 ^a^	10.88 ^b^	11.12 ^ab^	0.11	0.004
PDW (%)	51.12	81.78	66.38	57.15	48.28	60.82	57.23	10.47	0.551

Note: Results are based on six replicates (*n* = 6); SEM denotes the standard error of the mean. Within each row, different superscript letters (a–c) identify means that differ at *p* < 0.05. The blood-cell abbreviations are WBC (white blood cell count), RBC (red blood cell count), HGB (hemoglobin), and HCT (hematocrit). Erythrocyte indices comprise MCV (mean corpuscular volume), MCH (mean corpuscular hemoglobin), and MCHC (mean corpuscular hemoglobin concentration). PLT denotes platelet count; PCT, plateletcrit; MPV, mean platelet volume; and PDW, platelet distribution width.

**Table 5:** Serum biochemical measurements across the dietary AFB_1_ treatments.

Items	Concentration of AFB_1_ (μg/kg)							SEM	*p*-Values
	0	5	10	20	50	100	200		
ALT (U/L)	14.72	14.54	14.90	14.00	15.60	14.72	14.54	0.50	0.480
AST (U/L)	348.68	309.67	376.67	401.25	396.38	494.58	454.12	48.80	0.170
ALP (U/L)	1589.0	1179.9	1637.7	1551.1	1573.8	1414.4	1904.4	205.41	0.356
TP (g/L)	23.70	23.09	22.73	23.47	26.01	25.92	25.10	1.16	0.250
ALB (g/L)	11.36	10.24	10.60	10.70	12.52	11.97	11.81	0.59	0.096
TG (mmol/L)	0.46	0.36	0.36	0.42	0.36	0.34	0.39	0.04	0.479
GLU (mmol/L)	11.03	10.85	11.11	10.81	10.57	10.69	11.27	0.48	0.951
UREA (mmol/L)	0.47	0.44	0.37	0.38	0.54	0.38	0.43	0.06	0.512
TBIL (µmol/L)	8.60	8.82	10.60	10.30	11.21	9.91	11.21	0.95	0.309
CREA (µmol/L)	8.01	9.30	6.38	8.55	12.19	10.00	11.11	1.52	0.170
IgA (g/L)	0.98	1.20	1.05	1.10	1.29	1.20	1.17	0.11	0.504
IgG (g/L)	5.29	7.23	7.78	7.21	7.30	7.27	6.45	0.72	0.219
IgM (g/L)	0.98	1.26	1.37	1.15	1.75	1.38	1.21	0.20	0.231

Note: Results are based on six replicates (*n* = 6); SEM denotes the standard error of the mean. Enzyme abbreviations are ALT (alanine aminotransferase), AST (aspartate aminotransferase), and ALP (alkaline phosphatase). TP denotes total protein; ALB, albumin; TG, triglycerides; GLU, glucose; UREA, urea; TBIL, total bilirubin; and CREA, creatinine. The immunoglobulins measured were IgA (immunoglobulin A), IgG (immunoglobulin G), and IgM (immunoglobulin M).

**Table 6:** Effects of dietary AFB_1_ exposure on intestinal morphology of broilers.

Items	Concentration of AFB_1_ (μg/kg)							SEM	*p*-Values
	0	5	10	20	50	100	200		
Jejunum									
VH (μm)	1101.91 ^a^	1062.13 ^a^	1082.42 ^a^	1072.67 ^a^	777.49 ^b^	744.13 ^b^	779.72 ^b^	92.46	0.011
CD (μm)	139.45 ^c^	157.11 ^abc^	135.52 ^c^	184.50 ^a^	131.76 ^c^	144.88 ^bc^	176.52 ^ab^	11.37	0.011
VH/CD	8.19 ^a^	6.71 ^ab^	8.73 ^a^	5.99 ^ab^	6.05 ^ab^	5.16 ^b^	4.62 ^b^	0.94	0.037
Ileum									
VH (μm)	619.87	496.92	569.32	605.44	725.65	543.50	577.19	43.75	0.091
CD (μm)	100.07	119.77	82.34	89.77	114.17	91.67	105.71	9.31	0.109
VH/CD	6.22 ^abc^	4.81 ^c^	8.10 ^a^	7.26 ^ab^	7.69 ^ab^	6.06 ^bc^	5.48 ^bc^	0.69	0.029

Note: Results are based on six replicates (*n* = 6); SEM denotes the standard error of the mean. VH denotes villus height and CD denotes crypt depth; VH/CD is their ratio. Within each row, different superscript letters (a–c) identify means that differ at *p* < 0.05.

## Data Availability

The original contributions presented in this study are included in the article/Supplementary Materials. Further inquiries can be directed to the corresponding author.

## Supplementary Materials

The following supporting information can be downloaded at: https://www.mdpi.com/article/10.3390/toxins18100414/s1, Figure S1. Effects of graded dietary AFB_1_ exposure on bile acid profiles in different intestinal and systemic compartments of broilers. Table S1. Renal histopathological severity in broilers exposed to graded dietary AFB_1_.
