# Supplementary material for: Purified Dietary Aflatoxin B1 Does Not Impair Broiler Growth Across a Broad Exposure Range but Elicits Subclinical Biological and Tissue Responses
Source: Toxins (Basel). 2026 Sep 27;18(10):414. doi: 10.3390/toxins18100414 (PMC13709375; doi:10.3390/toxins18100414)
Supplement: Supplementary file 1 [file toxins-18-00414-s001.zip › toxins-4568222-supplementary.pdf]

toxins

MDPI

Supplementary Materials: Purified Dietary Aflatoxin B_1 Does Not Impair Broiler Growth across a Broad Exposure Range but Elicits Subclinical Biological and Tissue Responses

Qiaomin Duan, Wanjun Zhang, Ying Liu, Sunlin Luo, Wenjun He, Wenjun Yang and Yiqiang Chen [1,2,*]

This supplementary material contains 1 figure and 1 tables.

 https://doi.org/10.3390/toxins18100414

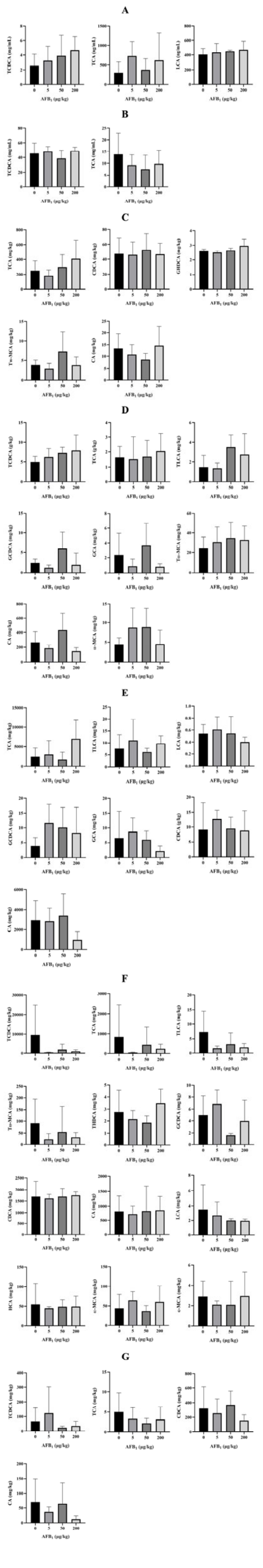

Figure S1. Effects of graded dietary AFB_1 exposure on bile acid profiles in different intestinal and systemic compartments of broilers. (A) Serum; (B) Bile; (C) Liver; (D) Duodenal contents; (E) Jejunal contents; (F) Ileal contents;(G) Colorectal contents of broilers were collected and analyzed for differential bile acid alterations in response to gradient AFB_1 treatment. n = 6. Significant differences versus the control group (0 μg/kg AFB_1), determined by one-way ANOVA followed by Duncan's multiple range test, are indicated as follows: * $P < 0.05$, ** $P < 0.01$, *** $P < 0.001$.

toxins

MDPI

Table S1. Renal histopathological severity in broilers exposed to graded dietary AFB_1.

Items	Concentration of AFB_1 (μg/kg)							H statistic	P-values
	0	5	10	20	50	100	200		
Lesion severity scores									
Renal tubular necrosis	0 (0–1)	2 (1–3)	0 (0–0)	0 (0–1)	4 (3–4)	4 (2–4)	0 (0–0)	17.376	<0.001
Cast formation	0 (0–1)	0 (0–0)	0 (0–0)	0 (0–0)	1 (0–1)	1 (0–1)	0 (0–0)	9.500	0.192
Brush border loss	0 (0–0)	0 (0–0)	0 (0–0)	0 (0–0)	0 (0–0)	0 (0–0)	0 (0–0)	—	—
Tubular dilation	0 (0–0)	0 (0–0)	0 (0–0)	0 (0–0)	0 (0–0)	0 (0–0)	0 (0–0)	—	—
Tubular basophilia	1 (1–1)	1 (0–1)	1 (1–1)	1 (1–1)	1 (1–1)	1 (1–1)	1 (1–1)	6.000	1.000
Inflammatory cell infiltration	1 (0–1)	0 (0–1)	0 (0–1)	1 (0–1)	0 (0–0)	0 (0–1)	1 (0–1)	4.444	0.844

Note: Values are presented as medians (minimum–maximum), with six birds per treatment, each from a different replicate cage. Renal lesions were evaluated by a single assessor blinded to dietary treatment using the following severity scale: 0, no lesion; 1, minimal; 2, mild; 3, moderate; and 4, marked. Each bird received a composite score for each lesion based on the examined microscopic fields; fields were not treated as independent biological replicates. AFB_1, aflatoxin B_1. Dietary concentrations denote nominal treatment levels. H denotes the Kruskal–Wallis test statistic. A dash indicates that no statistical test was performed because all scores were identical.Overall severity differences were evaluated using permutation-based Kruskal–Wallis tests. P values were adjusted using the Holm procedure across the four lesion endpoints with variable scores.
